# The Right Hemisphere Planum Temporale Supports Enhanced Visual Motion Detection Ability in Deaf People: Evidence from Cortical Thickness

**DOI:** 10.1155/2016/7217630

**Published:** 2016-01-14

**Authors:** Martha M. Shiell, François Champoux, Robert J. Zatorre

**Affiliations:** ^1^Montreal Neurological Institute, McGill University, Montreal, QC, Canada H3A 2B4; ^2^International Laboratory for Brain, Music, and Sound Research (BRAMS), Montreal, QC, Canada H2V 4P3; ^3^Centre for Research on Brain, Language, and Music (CRBLM), Montreal, QC, Canada H3G 2A8; ^4^École d'Orthophonie et d'Audiologie, Université de Montréal, Montreal, QC, Canada H3N 1X7; ^5^Centre de Recherche Interdisciplinaire en Réadaptation du Montréal Métropolitain, Institut Raymond-Dewar, Montreal, QC, Canada H2L 4G9

## Abstract

After sensory loss, the deprived cortex can reorganize to process information from the remaining modalities, a phenomenon known as cross-modal reorganization. In blind people this cross-modal processing supports compensatory behavioural enhancements in the nondeprived modalities. Deaf people also show some compensatory visual enhancements, but a direct relationship between these abilities and cross-modally reorganized auditory cortex has only been established in an animal model, the congenitally deaf cat, and not in humans. Using T1-weighted magnetic resonance imaging, we measured cortical thickness in the planum temporale, Heschl's gyrus and sulcus, the middle temporal area MT+, and the calcarine sulcus, in early-deaf persons. We tested for a correlation between this measure and visual motion detection thresholds, a visual function where deaf people show enhancements as compared to hearing. We found that the cortical thickness of a region in the right hemisphere planum temporale, typically an auditory region, was greater in deaf individuals with better visual motion detection thresholds. This same region has previously been implicated in functional imaging studies as important for functional reorganization. The structure-behaviour correlation observed here demonstrates this area's involvement in compensatory vision and indicates an anatomical correlate, increased cortical thickness, of cross-modal plasticity.

## 1. Introduction

When an individual is deprived of a sensory modality, the other senses can compensate for the loss with behavioural enhancements. This effect has been demonstrated in both deaf and blind humans, as well as in animal models of sensory deprivation (for a review, see [1, 2]). Generally, the sensory enhancements that occur after deprivation are attributed to the extra processing power that is afforded by the recruitment of the deprived sensory cortex, which is thought to reorganize to support the enhancement. Support for this relationship between enhanced sensory behaviour and cross-modal processing comes from human research on blindness, where enhanced performance on various tasks correlates with task-related activity [3–7], and cortical thickness [8] of visual regions in the occipital cortex. This relationship has also been demonstrated in congenitally deaf cats, where enhancements to visual motion detection and peripheral localization are abolished when the cat's auditory cortices are deactivated [9, 10]. While the evidence for this relationship is convincing, no research to date has established a direct connection between cross-modal plasticity and enhanced sensory behaviour in deaf people.

In deaf people, research on enhanced sensory behaviour and cross-modal plasticity has progressed mostly independently. In terms of sensory compensation, much research has focused on the role of vision. While some behavioural enhancements have been attributed to changes in visual attention (for a review, see [11]), others appear to be due to changes to basic sensory processing. These include enhancements to motion detection [12], discrimination of the angle of motion direction [13], a larger field of view [14, 15], and faster reaction times to visual stimuli [16–18], with a possible bias for peripheral visual fields [19, 20] (but see [17, 18]). Some of these behavioural enhancements may be supported by changes to both the peripheral and early cortical components of the visual system. For example, visual field area in deaf people correlates with neural rim area on the retina, denotative of increased retinal ganglion cells, and changes to the retinal neural fiber layer distribution [15]. Additionally, reaction times for target detection correlate with early event-related potentials in the visual cortex [17]. However, none of these behavioural enhancements have been directly associated with plasticity in the auditory cortex.

In terms of cross-modal plasticity for visual processing, multiple functional neuroimaging studies have identified visually evoked activity in the auditory cortices of deaf people, especially in response to stimuli that evoke visual motion, such as moving dots [21–24], gratings [25], and hands and/or lips [23, 26, 27]. In early-deaf people, this activity consistently occurs in the right hemisphere planum temporale and adjoining superior temporal gyrus [21–27]. The left planum temporale [25, 26] and primary auditory cortex [21, 22] also show activity in response to motion versus static stimuli, although these regions are not activated in every study [23, 26, 27]. While these studies clearly demonstrate the responsivity of the deprived sensory cortex to the nondeprived stimuli, they do not assess its association with enhanced sensory performance, as has been done in the human blind population with correlation and regression analyses (e.g., [3, 8]) and in deaf cats by manipulation of cortical function [9, 10]. Testing the relationship between the auditory cortex and vision is necessary to demonstrate that cross-modal reorganization in deaf people supports enhanced visual abilities [28].

In the current study, we hypothesized that compensatory visual enhancements in deaf people are supported by plasticity in auditory cortex. Based on parallel research questions in the blind [8], we reasoned that if a cortical region supports sensory enhancement in the deaf, then its cortical thickness will vary in relation to behavioural performance. Although much previous research has examined anatomical changes in the deaf brain, results have concentrated on changes that are associated with sensory deprivation [29–37] rather than compensatory plasticity. In auditory regions, these changes include decreased white matter volume [30, 33, 34, 38] and white matter integrity, as measured by diffusion-weighted MRI [29–31, 35]. In contrast, grey matter volume in auditory regions appears to be preserved after deafness [33–38]. Few studies have examined cortical thickness, and no changes have been documented between deaf and hearing adults in auditory regions with this measure [35]. Given the lack of evidence for atrophy of grey matter after deafness, we expected that cortical thickness might capture compensatory plasticity, rather than disuse-related atrophy.

To test our hypothesis, we used visual motion detection thresholds as a gauge for enhanced visual abilities, based on evidence for improved performance in the deaf as compared to hearing on this task [12]. With T1-weighted magnetic resonance imaging, we measured cortical thickness and tested for a correlation with behaviour in eight regions of interest (ROIs): the planum temporale (PT), Heschl's gyrus and sulcus (HGS), the middle temporal area (MT+), and the calcarine sulcus, bilaterally. Our primary prediction was that the cortical thickness of the right PT would correlate with enhanced visual abilities, given its consistent involvement in cross-modal processing of visual motion. Based on mixed results from previous research, we also explored the involvement of the left PT and bilateral primary auditory cortex, located within HGS. Finally, in addition to auditory ROIs, we considered the possibility that enhanced visual motion detection in deaf people is supported by changes to the visual system rather than, or in addition to, cross-modal processing in auditory regions. This consideration was inspired by previous research that demonstrates a correlation between visual ability and activity in the early visual system after deafness [17] and increased activity in MT+ for peripheral visual stimulation [39, 40]. As such, we included the calcarine sulcus, which encompasses primary visual cortex, and the motion processing area MT+.

## 2. Materials and Methods

The experiment was approved by the Research Ethics Board at the Montreal Neurological Institute and all participants gave written informed consent. A sign language interpreter was present throughout all testing sessions to translate (either* Langue des Signes Québécoise* or American Sign Language) between the experimenter and participant.

### 2.1. Participants

Eleven bilaterally, profoundly, and early-deaf people (5 men and 6 women; mean age = 28.2 years old; age range = 21–37 years old) participated in the study. All participants took part in an earlier study in our laboratory, which identified enhanced visual motion detection thresholds in deaf people [12]. Ten participants reported congenital deafness and one became deaf at six months of age due to meningitis. Two participants confirmed that their deafness was hereditary, and the remaining eight had unknown or unconfirmed etiologies. We used standard pure tone audiometry to measure monaural hearing thresholds in both ears of each deaf participant, confirming a hearing loss of greater than 90 dB at 500, 1000, 2000, and 8000 Hz in all participants but four, who were able to sense 500 Hz at 80 or 85 dB. Six participants were native sign language users who had typical language acquisition through early-life interaction with deaf family members, and five participants learned sign language in school around the age of five years and used a combination of signed French, home signs, and gestures to communicate prior to this. All participants used sign language as their primary language of communication once learned and used hearing aids during their childhood but stopped during their adolescence or earlier.

### 2.2. Visual Motion Detection Thresholds 

Threshold measures for visual motion detection were taken from our earlier study, and the details of the psychophysical procedure have already been published [12]. We used a two-alternative forced-choice procedure, in which participants maintained central fixation while viewing two simultaneously presented sinusoidal gratings (grating size: 6° × 6°, spatial frequency: 0.33 cycle/°, and Michelson contrast: 50%). The gratings were presented for 500 ms in the left and right visual fields, centered at −10° and +10°. In each trial, one of the two gratings was randomly selected to move while the other remained stationary, and participants were instructed to indicate, by button press, which of the two gratings was moving and to guess if uncertain. The speed of the motion varied according to a one-up one-down adaptive staircase procedure, with a 1 : 3 weighting in step size [41]. Eye movements were monitored with an Eyelink 1000 eye tracker (SR Research, Mississauga, ON, Canada), and trials were discarded from the staircase if fixation was broken. The staircase terminated after 15 reversals, which were averaged to give the threshold measure for that run. A run was discarded if the participant broke fixation in more than 18% of the trials (representing 2 standard deviations above the mean number of times that fixation was broken across all participants and runs). Participants completed 8 runs, and the median threshold across these runs was used as the final threshold measure.

### 2.3. MRI Acquisition

Scanning occurred at the McConnell Brain Imaging Centre of the Montreal Neurological Institute. We used a 3-T Siemens Trio Scanner with a 32-channel head coil to acquire T1-weighted MPRAGE scans (1.0 × 1.0 × 1.0 mm^3^ resolution, 176 slices, 256 × 256 matrix, and repetition time/echo time = 2300/2.98).

### 2.4. MRI Preprocessing

We used the Freesurfer Image Analysis Suite (http://surfer.nmr.mgh.harvard.edu/) to parcellate the regions of interest and automatically calculate cortical thickness across the brain. The details of this procedure are described in previous publications. In brief, the steps include removal of nonbrain tissue [42], intensity normalization [43], tessellation of grey and white matter borders, automated topology correction [44, 45], surface deformation [46, 47], surface inflation [48], and registration to a spherical atlas [49].

### 2.5. Selection of ROIs

Each brain surface was automatically parcellated into 56 regions in each hemisphere, according to the Destrieux atlas [50–52]. From this atlas, we extracted the mean cortical thickness in the PT, HGS, and the calcarine sulcus, bilaterally. Cortical thickness of MT+ was extracted via Freesurfer's built-in probabilistic map.

In previous research, cross-modal activations of the PT in deaf people typically include portions of the laterally adjoining posterior superior temporal gyrus [21–23, 25–27]. The expansiveness of these activations is not surprising, considering that functional activations of the PT in general are not constrained by the gross anatomical borders of this region [53], which are in any case often difficult to identify [54]. The spatially extensive activity of the PT is consistent with the fact that the cytoarchitectonic fields of this area also extend into adjacent areas, including parietal operculum, superior temporal sulcus, and supramarginal gyrus [55]. With this in mind, we chose to expand the borders of our planum temporale ROI by five vertices, increasing the surface area from 532.7 to 950.3 mm^2^ (in the Freesurfer standard space). In order to distinguish this ROI from the standard planum temporale output of Freesurfer, we will herein refer to it as the planum temporale region (PTR).

### 2.6. Analysis

For each of our eight ROIs, we tested for a Pearson partial correlation between visual motion detection thresholds and mean cortical thickness with age as a covariate. This covariate was included based on evidence that both motion detection ability [56] and cortical thickness [57] decline with age during adulthood. Specifically, a linear decrease of 10.5% has been documented in the cortical thickness of the superior temporal cortex from eight to thirty years of age [57], and a linear increase in coherent motion detection thresholds from nineteen to ninety-two years of age [56]. Additionally, in an earlier study from our lab that used the same task as used here to measure visual motion detection thresholds in 36 hearing and deaf adults, thresholds increased from twenty-one to fifty-six years of age (*r* = 0.48; *p* = 0.003; unpublished statistic with data from [12]). Based on this evidence, we reasoned that age may explain some of the variance in our hypothesized relationship between cortical thickness and visual motion detection thresholds, and thus its inclusion has the potential to strengthen the predicted effect.

For our primary hypothesis concerning the right PTR, we considered correlations where *p* < 0.05 (two-tailed) to be significant. We made no prediction about the direction of the relationship, given that both increased [8] and decreased [58] cortical thickness have been associated with cross-modal plasticity in previous research in the blind. The remaining ROIs were exploratory, with mixed support for their involvement in cross-modal activity (see Introduction), and thus we applied a Bonferroni correction for multiple comparisons, where we considered correlations of *p* < 0.007 (two-tailed) to be significant. This threshold is equal to the *p* < 0.05 threshold used for our primary hypothesis, divided by 7, which is the number of exploratory comparisons that we pursued.

We also carried out a vertex-wise analysis within our ROIs in order to explore whether or not specific subregions of these areas were related to our behavioural measure. This was particularly relevant to the case of the PTR, which is thought to consist of several functional subregions [53], and the calcarine sulcus, where effects might be specific to the areas that represent peripheral visual space [59]. For this analysis, we smoothed the data with a 15 mm FWHM Gaussian kernel and performed a vertex-wise regression of visual motion detection thresholds to generate a *Z*-statistic map and considered all vertices within our ROIs that had a probability of *p* < 0.01, uncorrected for multiple comparisons. It should be noted that this second analysis differs from the first because it strived to localize changes within the ROIs, rather than identify which ROIs correlated with cortical thickness.

With this vertex-wise analysis we uncovered a subregion within the right PTR that varied in cortical thickness according to visual motion detection thresholds (see Results). To further characterize the location of this subregion, we expanded it to include all adjacent vertices that passed a threshold of *p* < 0.01 uncorrected, unconstrained by the boundaries of our ROI. This was necessary to ensure that our result was primarily within the PTR, rather than an overlap from a cluster centered on an adjacent region.

In an earlier fMRI study from our lab [25], we identified an area centered in the posterior superior temporal gyrus where deaf individuals showed activity in response to visual motion. Five participants from the current study took part in this earlier experiment, which tested early-deaf people with varying degrees of residual hearing [25]. We wanted to assess if this previous fMRI result could be the functional equivalent of the current study's anatomical result. To do so, we transformed the results from the previous study into the average surface space and calculated the percentage overlap of the two regions. Finally, in order to fully describe our effect, we compared its mean cortical thickness to that of 11 hearing controls from our earlier dataset [25] that were selected to match the age and gender distribution of the current study. The cortical thickness of the hearing control participants was measured with identical imaging and analysis parameters to those of the current study, described above.

## 3. Results

**Figure 1:**
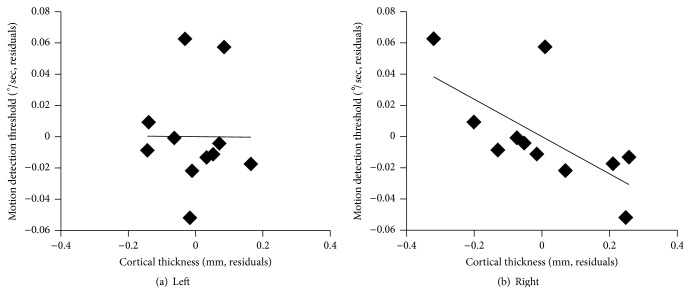
Partial correlation between mean cortical thickness in the left (a) and right (b) PTR and visual motion detection thresholds in deaf people after controlling for age. In the right PTR but not in the left, cortical thickness correlated with visual motion detection thresholds.

Our primary hypothesis was that the cortical thickness of the right PTR would correlate with visual motion detection thresholds. We found a negative partial correlation (Figure 1, *r* = −0.66, *p* = 0.026, two-tailed, *n* = 11, degrees of freedom = 8) after controlling for participant age. Greater cortical thickness of this area was correlated with enhanced visual motion detection thresholds. This effect was absent if age was removed as a covariate. There was no correlation between visual motion detection thresholds and cortical thickness in any other region (left PTR: *r* = −0.01, *p* = 0.987; left HGS: *r* = 0.43, *p* = 0.218; right HGS: *r* = −0.51, *p* = 0.131; left MT+: *r* = −0.32, *p* = 0.21; right MT+: *r* = 0.27, *p* = 0.22; left calcarine sulcus: *r* = 0.42, *p* = 0.230; right calcarine sulcus: *r* = −0.14, *p* = 0.693). One-tailed paired-sample Student's* t*-test on the Fisher-transformed correlation coefficients from the right and left PTR indicated that the correlation in the right PTR was stronger than that in the left (Figure 1, *t* = 3.1, *p* = 0.01). Mean cortical thickness values for each participant in each ROI are listed in Table 1.

**Figure 2:**
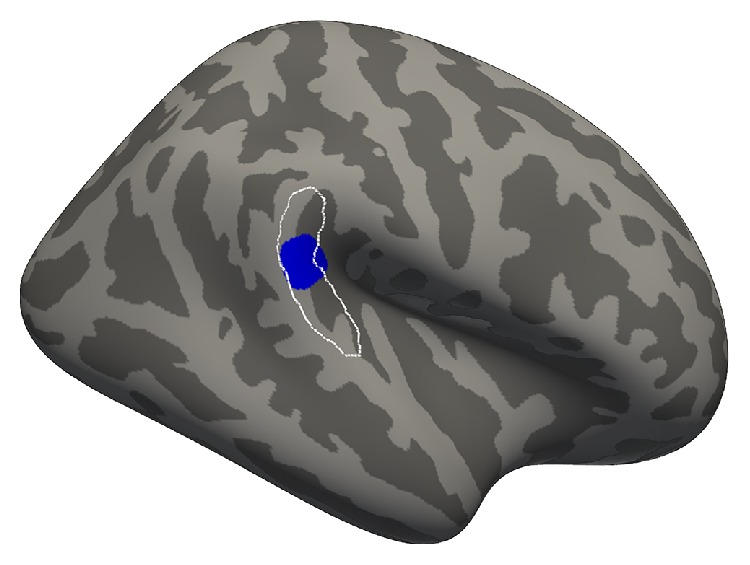
Visual motion detection thresholds in the right PTR predict cortical thickness (blue region). The region of the effect was first identified in the right PTR ROI (white outline) according to our* a priori* hypothesis and then expanded to include all vertices at *p* < 0.01, in order to explore its location when unbounded by the ROI.

**Figure 3:**
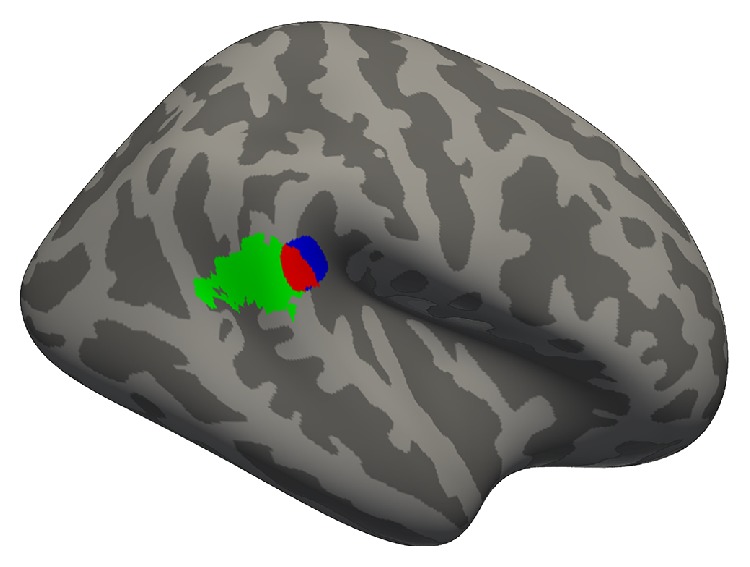
Overlap (red) between the region in the right PTR where cortical thickness predicts visual motion detection thresholds (blue + red) and a region of visual motion-related cross-modal activity in deaf people from Shiell et al. (2015) [25] (green + red).

**Figure 4:**
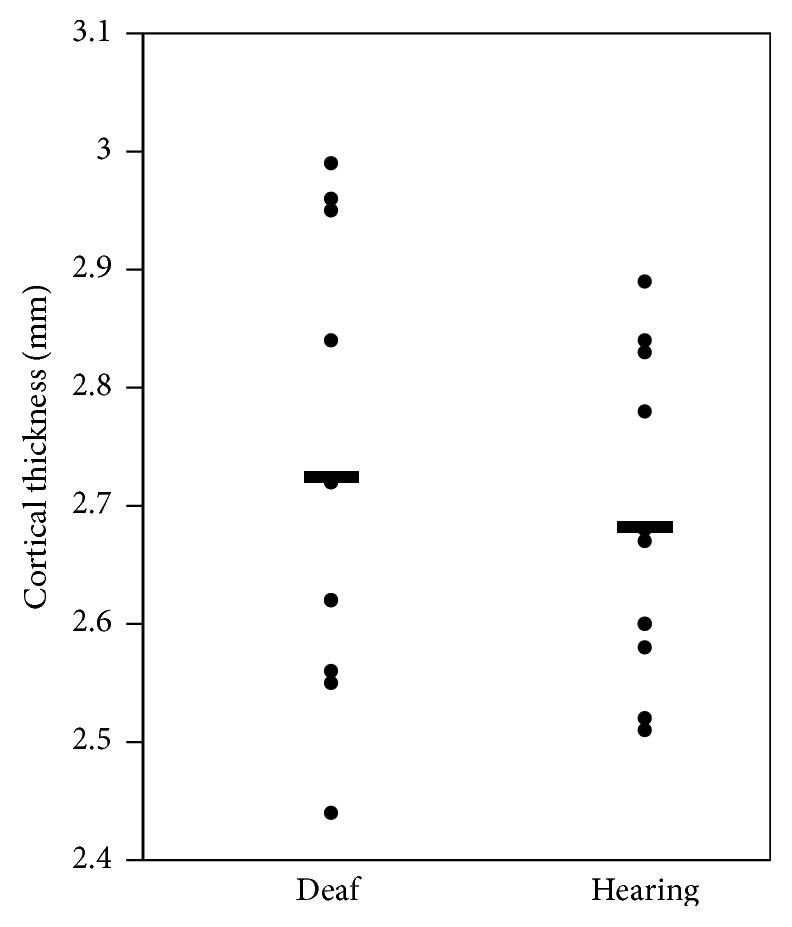
Mean cortical thickness of the right hemisphere planum temporale region. Horizontal bars indicate group means. Cortical thickness in the deaf group was not different from a hearing group matched for age and gender, taken from Shiell et al. (2014) [12].

In the vertex-wise regression within the ROIs, we uncovered a subregion of 238.5 mm^2^ within the right PTR, where cortical thickness predicted behavioural performance (*p* < 0.01, uncorrected for multiple comparisons, maximum *Z*-statistic = −2.72 at MNI152 coordinates 63, −37, 17). When unbounded by the PTR ROI, this subregion expanded to 273.6 mm^2^ (*p* < 0.01, uncorrected for multiple comparisons) and remained centered in the PTR ROI (Figure 2). Nearly half of this cluster (47%) overlapped with a region that demonstrated cross-modal activity in deaf people in a previous fMRI experiment from our lab (Figure 3) [25]. This expanded region had an average cortical thickness of 2.73 mm (±0.19 mm standard deviation), which did not differ from hearing controls (Figure 4, mean = 2.68 mm, standard deviation = 0.14 mm; *t* = 0.623, *p* = 0.54).

## 4. Discussion

Consistent with our prediction, we found that cortical thickness in the right PTR correlates with enhanced performance on a visual motion detection task in early-deaf people: Greater cortical thickness was associated with better thresholds, when age was controlled for (Figure 1). Our finding supports the idea that compensatory visual enhancements are supported by cross-modal structural plasticity after deafness, establishing the first direct evidence for this relationship in deaf humans. The finding is consistent with prior data because it was detected in a region where cross-modal activations in deaf people have been reported in previous studies [21–24, 26, 27], including one from our lab [25] (Figure 2).

A direct comparison of the regions of effect in our current and previous studies [25] shows a partial overlap, with the current study's effect localized more medially, centered on the superior bank of the superior temporal gyrus rather than on its lateral surface (Figure 3). The relative closeness of these regions of effect supports the idea that they represent corresponding functional and anatomical cross-modal plasticity, particularly in the subregion where they overlap. However, we cannot draw definitive conclusions from their comparison, as the studies used different participant groups and image processing strategies. Regardless of their correspondence with one another, both results implicate posterior regions of the superior temporal lobe, confirming a role of this area in cross-modal reorganization after deafness.

The structure-behaviour relationship uncovered here is consistent with findings from congenitally deaf cats, which show that cross-modal activity supports enhanced visual abilities. In deaf cats, motion detection thresholds were associated with activity in a region that extends dorsally from primary auditory cortex, known as the auditory dorsal zone (DZ). Given their covariation (in activity and thickness, resp.) with visual motion detection thresholds across species, we propose that the cat's DZ and the current study's region of effect may be similarly reorganized after early deafness. One prediction from this idea is that the cortical thickness of the DZ and motion detection thresholds of deaf cats will correlate. A comparison of cortical volume of the DZ in deaf and hearing cats found no global differences; however, neither cortical thickness nor the potential correlation between levels of activity and visual ability was examined [60].

Our finding mirrors what has been identified in the blind population, where increased cortical thickness in the occipital lobe was associated with enhanced performance on pitch and melody discrimination tasks [8]. However, anatomical MRI research in blind people differs from the deaf, in that the blind show widespread differences from the sighted in the cortical thickness of visual areas [61–63]. In contrast, anatomical comparisons between deaf and hearing adults report null differences in grey matter measures of auditory regions in the temporal lobe for either cortical thickness [35] or volume [33, 34, 36] and likewise for cortical volume in deaf and hearing cats [60]. Similar to this previous work, we found no global difference between deaf and hearing in the cortical thickness of our region of effect (Figure 4). Despite this lack of difference between deaf and hearing groups, we have confirmed that cortical thickness of auditory regions in humans is indeed altered after deafness and that these alterations are identified only when examined in the context of enhanced visual behaviour and age. Our finding suggests that many different factors influence the cortical thickness of the planum temporale region, such that no global difference arises between deaf and hearing, but when relevant variables can be identified, such as visual motion detection abilities, then some of the variance can be accounted for. This complexity may reflect the fact that cortical thickness captures the interaction of numerous different cellular-level mechanisms, which can reflect both adaptive and nonadaptive plasticity (for a review, see [64]). In the case of cross-modal plasticity after deafness, recent research on deaf cats demonstrates one possible adaptive mechanism: in a cross-modally active subregion of auditory cortex, early-deaf cats show increased dendritic spine density as compared to hearing cats [65]. Speculatively, this cellular-level change may occur in tandem with increased axonal branching, which could in turn increase cortical thickness.

The relationship between cortical thickness and visual motion detection was not found in the left PT, nor in either hemisphere's periprimary auditory areas on HGS, the motion processing area MT+, or primary visual cortex in the calcarine sulcus. Our vertex-wise regression analysis within the ROIs helps mitigate the risk that only subregions of these areas had an effect. However, we cannot rule out the possibility that an effect may have been found with a more individualized definition of these ROIs, such as by mapping the retinotopy of V1 and using only regions that represent peripheral visual space or by defining MT+ within each participant via a functional localizer. As they are, our results provide no evidence that plasticity in these regions is related to enhanced visual motion detection.

Interestingly, in our study the correlation between visual ability and cortical thickness occurred exclusively in the right hemisphere. This implies that cross-modal plasticity for visual motion in the deaf may be lateralized and is consistent with suggestions from previous research where the cross-modal activity for moving stimuli was exclusive to the right hemisphere [21–24, 27] or appeared stronger in the right than left [25, 66]. This lateralization is in contrast to deaf cats, where only bilateral deactivation was effective at inhibiting enhanced behaviour [9], and highlights the relevance of cross-species comparisons. The right hemisphere is widely believed to be specialized for spatial processing, an idea inspired by this hemisphere's role in spatial neglect disorders (for a review, see [67]). Key to these disorders is the right temporoparietal junction (TPJ), an area just posterior to our region of effect. The TPJ is implicated in reorienting attention to behaviourally relevant sensory targets [68]. As part of this attention module, activity that is related to auditory stimulus-driven attention is localized to the anterior portion of the TPJ, extending into the posterior superior temporal lobe [69]. Given our region of effect's proximity to these functions, we suggest that our effect may reflect reorganization of an area involved in auditory sensory reorienting. Following this idea, the enhancement of visual motion detection in deaf people may be due to a global enhancement to detect changes in the environment for the purpose of sensory reorienting.

There is also a region in the posterior PT that shows sensitivity to auditory motion stimuli (e.g., [70]), which has led to the suggestion that auditory motion sensitivity could be coopted to support visual motion sensitivity after deafness [21, 22]. This explanation may be complementary to our suggestion of reorganized sensory reorienting, as motion sensitivity in this area may interact with sensory reorienting. Both the sensory reorienting and motion processing interpretations are consistent with evidence that cross-modal reorganization exploits the homology of functions across different sensory modalities [9, 10].

Our investigation is limited within the deaf population to early-deaf sign language users with minimal hearing aid use. Previous research indicates that the age of onset of deafness [71], duration of deafness [72], early language experience [27, 73–75], and duration of hearing aid use [25] can each affect neural organization after deafness. Thus, future research needs to investigate how our results generalize to different deaf populations, such as those with residual hearing or adult-onset deafness. Importantly, since all of our participants used sign language as their primary mode of communication, we cannot separate the effects of deafness and language use. Although there is some evidence that sign language experience (versus oral language experience) alters neuroanatomy (e.g., [38]), we think it is unlikely that our effect is due to sign language alone, considering its parallelism with research in deaf cats [9] that have no language experience. Future research should also investigate whether motion detection thresholds are related to structural variation in hearing people. Relationships between visual ability and grey matter structure of the visual cortex have been demonstrated in the typical hearing population in previous research (e.g., [76]).

Since we only examined one measure of enhanced vision in deaf people, future research may also investigate whether or not other behavioural enhancements are related to cross-modal plasticity. If, as we have proposed, the involvement of our region of effect has to do with changes to the cortical system for sensory reorienting, then performance on tasks that involve target detection at unattended locations [77], such as those where deaf people show an advantage in reaction times [16–20], should show a similar relationship with cortical thickness in the right PTR. Since these sensory enhancements may also reflect changes to early visual processing [15, 17], an important step will be to understand how plasticity affects the interactions between auditory and visual cortices.

## 5. Conclusions

This research provides evidence that the right posterior superior temporal cortex reorganizes to support enhanced visual motion detection abilities in early and profoundly deaf people and that this plasticity is marked by increased cortical thickness.

## Figures and Tables

**Table 1:** Dataset for testing the correlation between cortical thickness and visual motion detection thresholds, controlling for participant age.

Participant	Age (years)	Motion detection threshold (deg./s)	Mean cortical thickness (mm)							
			Left hemisphere				Right hemisphere			
			CS	MT+	HGS	PTR	CS	MT+	HGS	PTR
1	30	0.23	2.23	2.33	2.83	2.5	2.19	2.45	2.84	2.72
2	26	0.17	1.98	2.34	2.5	2.47	2.06	2.49	2.57	2.72
3	24	0.20	1.85	2.39	2.53	2.28	1.88	2.52	2.37	2.55
4	23	0.25	2.04	2.32	2.79	2.39	2.03	2.46	2.24	2.44
5	34	0.15	2.04	2.28	2.53	2.26	2.21	2.26	2.54	2.56
6	21	0.18	2.03	2.30	2.62	2.41	1.98	2.36	2.54	2.84
7	32	0.15	1.97	2.31	2.55	2.44	2.08	2.43	2.75	2.96
8	25	0.13	1.97	2.45	2.68	2.4	2.15	2.40	3.17	2.99
9	25	0.17	2.04	2.50	2.85	2.58	2.33	2.53	2.78	2.95
10	37	0.14	1.76	2.36	2.55	2.48	2.03	2.35	2.61	2.62
11	33	0.16	1.96	2.21	2.49	2.35	2.14	2.35	2.44	2.62

CS, calcarine sulcus; MT+, middle temporal area; HGS, Heschl's gyrus and sulcus; PTR, planum temporale region.
